# An Automated Electrochemistry Platform for Accelerating the Characterization of Enzymatic Electrochemistry

**DOI:** 10.1021/acselectrochem.6c00095

**Published:** 2026-04-29

**Authors:** Michael A. Pence, Zachary A. Nguyen, Luke G. Kays, Dylan G. Boucher, Joaquín Rodríguez-López, Shelley D. Minteer

**Affiliations:** † 14717 Missouri University of Science and Technology, Department of Chemistry and Kummer Institute Center for Resource Sustainability, Rolla, Missouri 65409, United States; ‡ University of Utah, Department of Chemistry, Salt Lake City, Utah 84112, United States; § University of Illinois Urbana-Champaign, Department of Chemistry and Beckman Institute, Urbana, Illinois 61801, United States

**Keywords:** automation, high-throughput, cyclic voltammetry, enzyme electrochemistry, glucose oxidase

## Abstract

Enzymatic electrochemistry harnesses the selectivity of enzymes to enable electrochemical applications spanning sensing, synthesis, and energy conversion. However, the sequential nature of electroanalytical experiments limits throughput, restricting the scale at which enzyme-electrode systems can be screened. Here we demonstrate the capabilities of an automated electrochemistry platform, eLab, to increase the throughput of enzymatic electrochemistry investigations. We used the eLab to collect over 10,000 cyclic voltammograms across a large parameter space consisting of two enzyme variants (promiscuous and wild-type glucose oxidase), 20 saccharide substrates, 21 concentrations, and four scan rates, with measurements being made all in triplicate. The expansive dataset enabled rapid identification of apparent outlier behavior of wild-type glucose oxidase toward glucose, which was confirmed to arise from oxygen sensitivity through targeted manual experiments. The promiscuous variant showed negligible oxygen sensitivity, a critical advantage for applications, such as enzymatic sensors, bioelectrosynthesis, and biofuel cells. Overall, this work demonstrates how automation can be applied to accelerate discovery in bioelectrochemistry.

## Graphical abstract

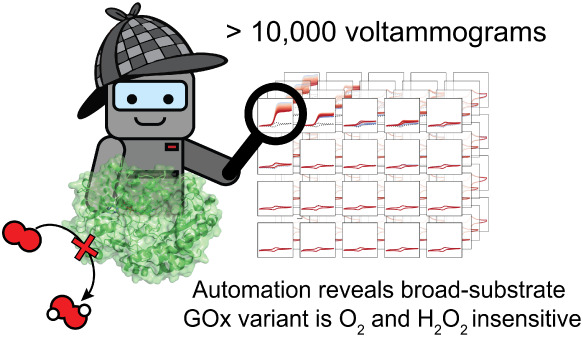

## Introduction

Enzymes are powerful catalysts that provide large rate enhancements for challenging reactions while maintaining high chemo-, regio-, and even enantioselectivity. Coupling enzymatic selectivity and activity with redox reactions driven at electrode surfaces via mediated electron transfer reactions enables applications that span sensing, synthesis, and energy conversion.^1–5^ Electrochemical methods are particularly insightful in the characterization of the redox behavior of oxidoreductases, enzymes which account for nearly one-third of all classified types in the Braunschweig Enzyme Database and are widely used in industrial applications.^6,7^ For example, monooxygenases such as cytochrome P450s are widely used in bio­(electro)­catalysis and serve as common starting points for directed evolution campaigns owing to their ability to activate difficult bonds such as C–H bonds for optimized non-native organic biocatalysis.^8–10^ Electroanalytical methods can provide information about the thermodynamics and kinetics of oxidoreductases, even at the single-enzyme level, making electrochemistry an excellent screening platform for this important class of biomolecules.^11–19^

Despite the promise of electrochemistry as a screening platform for enzymatic behavior, there are major throughput limitations. The sequential nature of electroanalytical experiments makes them particularly time-consuming and lower throughput than standard techniques for enzyme characterization such as 96-well plate colorimetric assays.^20,21^ Automated electrochemistry platforms promise to increase the throughput and reproducibility of electroanalytical characterization,^22^ offering a pathway toward incorporating electrochemistry as a screening tool in modern enzymology workflows.^23^ Additionally, the reduced human effort afforded by automated electrochemical characterization enables titration-like experiments with high-density sampling over a parameter space to reveal nuanced trends that would be missed with fewer experimental data points.^24,25^ There have been a number of efforts in automating electrochemistry across a variety of fields ranging from sensing,^26–28^ electrocatalysis,^29–31^ and energy storage,^32–37^ to electrosynthesis.^38–42^ However, there have been significantly fewer efforts in automating studies of enzymatic electrocatalysis, with existing studies having only explored single enzyme-substrate combinations.^43,44^

Figure 1a depicts possible use cases for automated and high-throughput electrochemical characterization in screening the interaction between enzymes and mediators, optimizing solution conditions for enzyme stability and activity, screening mutants to understand structure-activity relationships, and evaluating enzyme activity across a substrate scope. Of these possible use cases, we were most interested in screening enzymatic activity across a broad range of substrates, as it is a key factor in evaluating enzyme performance in electrochemical sensors, electrosynthesis, and biofuel cells. Understanding the promiscuity of an enzyme toward off-target compounds also provides an exploratory seed for directed evolution campaigns toward new reactivity.^23,45^

**Figure 1:**
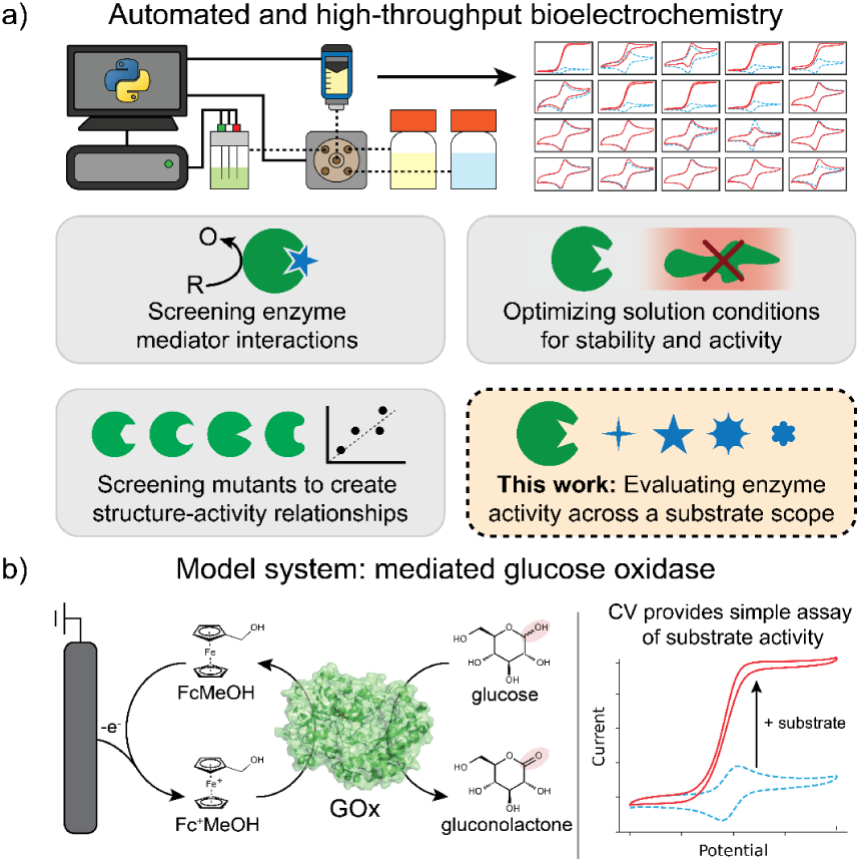
(a) Cartoon schematic of the eLab platform used in this work (detailed schematic shown in Figure S1) and an overview of the applications of automated electrochemistry to enzymatic systems and (b) the system studied in this work; mediated electrocatalysis of glucose oxidase (GOx). Here, hydroxymethylferrocene (FcMeOH) is oxidized at the electrode surface, subsequently oxidizing the cofactor of GOx via two successive single-electron transfers. Fully oxidized GOx then turns over the substrate, generating an enhanced current. The observed current enhancement in cyclic voltammetry (CV) upon the addition of substrate provides a simple assay of substrate activity.

To enable accelerated studies of enzyme activity across a large substrate scope, here we employed the eLab automated electrochemistry platform, which has been previously used for electroanalytical studies of mediated electrocatalysis.^25,46^ The eLab platform integrates a syringe pump and a multichannel fluidic valve to automate complex solution-handling operations, including mixing reagents for analysis, cleaning the electrochemical cell between tests, and directing solutions to waste. We chose glucose oxidase (EC 1.1.3.4) as a proof-of-concept enzyme, as it has been extensively characterized in the literature, shows negligible fouling of electrode surfaces, and has broad-substrate variants with expanded substrate scopes. Figure 1b depicts the mechanism of mediated electrocatalysis with glucose oxidase as well as the expected voltammetric response upon substrate addition. The addition of substrate enhances the voltammetric current by regenerating the initial form of the redox mediator. The steady-state response depicted is due to the kinetically controlled reaction confining the diffusion layer to a constant thickness, resulting in steady-state mass transport and a sigmoidal potential-current curve. Previous efforts from the Minteer group have explored the reactivity of the broad-substrate variant, finding that it enables the oxidation of di-, tri-, and polysaccharides.^47^ The broad-substrate variant allowed for biofuel cells to operate with complex mixtures rich in sugar, achieving power densities of up to 80 μW/cm^–2^ when operating with half-and-half milk as the anodic fuel. Here, we used our automated electrochemistry platform to screen both the wild-type glucose oxidase (GOx) and the broad-substrate variant (bGOx) across 20 different sugars, demonstrating the capabilities of the automated electrochemistry platform, eLab, for bioelectrochemical applications.

## Experimental Section

### Materials

bGOx (glucose oxidase (broader substrates) (lot no. N0151331R)) was purchased from Amano Enzyme Inc. GOx (glucose oxidase (type VII, lyophilized powder, ^3^100,000 units/g)), d-glucose ^3^97.5%, 2-deoxy-d-glucose ^3^98%, d-mannose ^3^99%, d-galactose ^3^99%, d-xylose 99%, d-glucosamine ^3^99%, d-maltose ^3^99%, d-lactose ACS reagent grade, d-raffinose ^3^99%, d-trehalose ^3^99%, d-fructose ^3^97%, methyl-d-glucoside 97%, sodium phosphate dibasic heptahydrate, sodium phosphate monobasic monohydrate, and ferrocene methanol (FcMeOH) 97% were purchased from Sigma-Aldrich. Myo-inositol ^3^99%, d-allose ^3^98%, d-arabinose ^3^99%, d-ribose ^3^97%, and d-tagatose ^3^99% were purchased from Chem-Impex International. d-sucrose ^3^97% and hydrogen peroxide 30 wt % solution in water were purchased from Fisher Scientific. l-glucose 98% and d-cellobiose 98% were purchased from Ambeed. Pt mesh and wire (99.99%) were purchased from Goodfellow Advanced Materials. Glassy carbon (GC) (3 mm) and saturated calomel electrode (SCE) were purchased from CH Instruments. Water (18 MΩ) was purified by using a Milli-Q Water Purification System.

### Preparing Stock Solutions

We prepared a 1 L stock solution of 100 mM potassium phosphate buffer solution (KPi) pH 6.5 using a mix of 5.874 g of Sodium Phosphate Dibasic Heptahydrate and 9.019 g of Sodium Phosphate Monobasic Monohydrate and then adjusted it to the desired pH. This buffer was then used to prepare the following stock solutions: 300 μM FcMeOH in KPi, 8.1 μM bGOx/GOx in KPi, and 300 mM substrate in KPi, with substrates varying from experiment to experiment. The substrate stock solutions were allowed to epimerize for 24 h in a 4 °C fridge prior to use in experiments.

### Automated Electrochemistry Platform

The automated electrochemistry platform in this work was an identical replicate of the platform described by Pence et al., consisting of an SY-08 syringe pump and an SV-07 multiport switching valve (Figure S1) controlled by the eLab library.^25^ Control of the potentiostat was carried out using the Hard Potato library.^48^ Aerobic peroxide production experiments were carried out using an automated stirring setup, which used an Arduino-based circuit to switch power to an IKA Big Squid stir plate (Figure S2).

### Electrochemical Experiments

All electrochemical experiments were performed with either a CH611E or CH660E series potentiostat (CH Instruments), which perform similarly for the purposes of the experiments in this work. Most electrochemical measurements were performed in a three-electrode configuration, with a 3 mm diameter glassy carbon working electrode, a platinum wire counter electrode, and a saturated calomel electrode (SCE) as the reference electrode. Measurements of hydrogen peroxide production were performed with a 2 mm diameter platinum working electrode. All electrochemical experiments were performed in 100 mM KPi (pH 6.5) as the supporting electrolyte. Anaerobic experiments were carried out in an argon-filled vinyl anaerobic chamber (Coy Laboratory Products) at <1 ppm of O_2_ in degassed buffer solution.

Substrate screen CVs were collected by sweeping oxidatively from −0.2 V to 0.6 V vs SCE and back at four scan rates (10, 25, 50, and 100 mV/s). Each experimental run followed an automated workflow: the cell was primed and filled with 2.7 μM enzyme in KPi, baseline CVs were collected, and the solution was removed and rinsed with DI water. A solution of 2.7 μM enzyme and 100 μM FcMeOH was then added, and baseline voltammograms were collected in the absence of a substrate. Subsequently, 300 μL aliquots of 2.7 μM enzyme, 100 μM FcMeOH, and 100 mM substrate were titrated into the cell, with CVs collected after each addition. The cell was then thoroughly rinsed with DI water, and the entire titration series was repeated in triplicate, with each replicate data point obtained from a separate titration following cleaning of the cell. Each substrate screen used approximately 12 mL each of the enzyme and mediator solutions.

## Results and Discussion

We investigated the scope of the 20 commercially available substrates shown in Scheme 1. The high-throughput nature of the measurement allowed us to extend this beyond previous studies of bGOx promiscuity,^47^ including monosaccharides such as 2-deoxy-d-glucose and d-mannose that have been reported to elicit a response from the wild-type GOx enzyme.^49^ Overall, 10,240 CVs were collected over the entire sample space explored in this work, resulting in ∼13 days of continuous experimentation in an automated fashion by the eLab platform. Such titrations could also be performed through chronoamperometric methods;^47^ however, this loses possible mechanistic information that can be gleaned from the potential-current curves. We note that there was manual intervention between each substrate-enzyme combination, where the enzyme solution was freshly prepared, and the working electrode was polished before the subsequent 8-hour automated run. However, the manual effort needed was a fraction of the actual continuous experimental time, allowing for passive data collection, during which the experimenter can focus on other endeavors. We estimate that if this work were conducted manually, it would have taken at least a month of continuous effort.

**Scheme 1:**
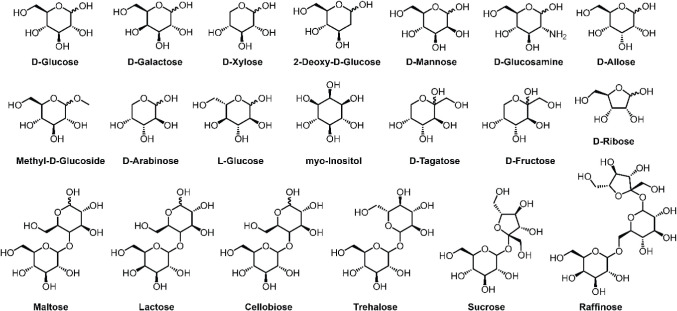
Structures for All Molecules in the Substrate Scope Explored in This Work, Consisting of a Wide Variety of Mono-, Di-, and Trisaccharides^a^

a Sugars studied include d-glucose, l-glucose, and 2-deoxy-d-glucose; the hexoses d-mannose, d-galactose, d-fructose, d-allose, and d-tagatose; the pentoses d-xylose, d-arabinose, and d-ribose; the amino and modified sugars d-glucosamine and methyl-d-glucoside; the disaccharides d-maltose, d-lactose, d-trehalose, d-sucrose, and d-cellobiose; the trisaccharide d-Raffinose; and the sugar alcohol myo-inositol.

Figure 2 depicts the data processing for our studies. The ratio between the CV plateau current in the presence of the substrate, *i*_*pl*_, and the peak current in the absence of the substrate, *i*_*p*_, for every scan rate measurement was calculated to compare titration experiments across different enzyme-substrate combinations. The lowest scan rate used in this work was 10 mV/s to ensure sufficient throughput, but this may not be appropriate for rigorous kinetic analysis when the kinetics of enzyme reactivity is such that a steady state cannot be reached (Figure S3). Using this current ratio method allows us to extract the substrate-dependent activity from the voltammetric data, making for simpler comparison and enabling extraction of observed rate constants for further kinetic analysis (Note S1, Figure S4), although accurate kinetic analysis is nontrivial for glucose oxidase.^50^

**Figure 2:**
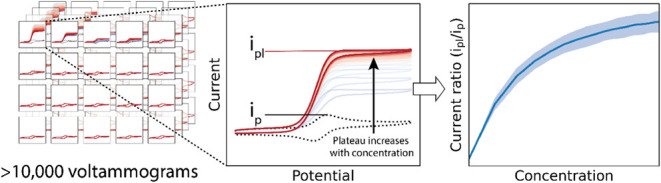
Schematic of the automated platform and data processing workflow, emphasizing the quantity of data produced (over 10,000 CVs). CVs were carried out across a combination of 2 enzyme variants, 20 substrates, 21 concentrations, and 4 scan rates with each condition screened in triplicate. Data was converted from CVs to current ratio versus concentration plots to better visualize concentration-dependent activities of the substrate.

Voltammetric responses were reproducible across three trials for both enzymes, and the measured CVs showed no impact of substrate diffusion, making peak-ratio analysis acceptable for comparison among experiments (Figures S5 and S6).^51^ We saw no evidence of electrode fouling during the automated experiment. Most notably, there is no sequence dependence when examining FcMeOH behavior across replicates (Figure S7), which would be a clear indicator of a film forming on the electrode, such as one from a denatured enzyme, and blocking electron transfer. Additionally, CV studies of representative sugars show no signal above the nonfaradaic background obtained in buffer, indicating that there is likely no surface passivation occurring from the direct electrooxidation of sugars (Figure S8). The few samples that showed significant deviation across the triplicate measurements (i.e., d-sucrose in Figure S5) were not sequence dependent, and deviations likely arose from solution handling, which can introduce bubbles that can adhere to the electrode and alter its effective area and resultant current. To further ensure the validity of using automated electrochemistry for this system, we ensured that CVs in the absence of substrate gave the expected current response for FcMeOH, with Randles–Ševčík analysis yielding a mean diffusion coefficient of 6.20 × 10^–6^ cm^2^/s with a standard deviation of 0.08 × 10^–6^ cm^2^/s (Figure S9). This value is similar to previously measured diffusion coefficient values of FcMeOH (∼7 × 10^–6^ cm^2^/s), validating that our automated platform is reliable for electroanalysis.^52,53^

Figure 3 shows the catalytic response as a function of concentration for bGOx and GOx. Current ratio – concentration plots are shown for 10 mV/s, as these give the most prominent response (Figure S10). The black dotted line is the current ratio in the absence of substrate, and curves that lie entirely below this line indicate negligible activity of the enzyme to the substrate. Only six monosaccharides gave a notable response for either enzyme: d-glucose, 2-deoxy-d-glucose, d-mannose, d-galactose, d-xylose, and d-glucosamine, although the activity trends differed between GOx and bGOx. The most prominent difference in substrate activities between the two enzymes was their response to the disaccharides. This is in line with previous observations, where di-, tri-, and even oligosaccharides such as starch were able to be oxidized by bGOx if they contained 1,4-glycosidic linkages but were unreactive with GOx. Interestingly, we saw a difference in the shape of the disaccharide response curves for d-lactose and d-cellobiose, both of which have β-1,4 glycosidic linkages. The origin of this apparent substrate inhibition remains unclear, as it was not observed in previous work.^47^

**Figure 3:**
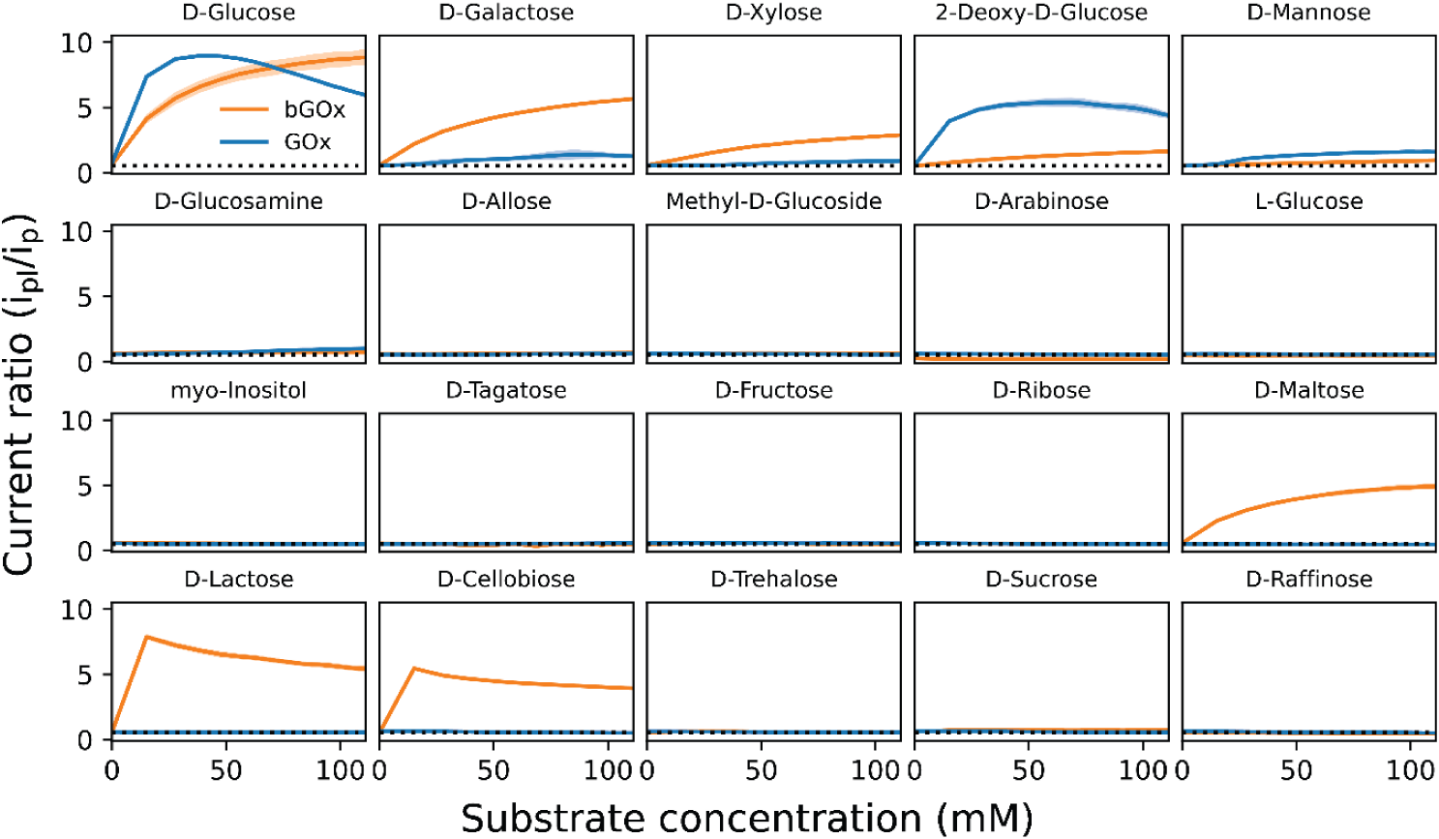
Results of electrochemical screening across the substrate scope. Data was processed as described in Figure 2, with plots showing the current ratio with and without substrate as a function of concentration. Error bars represent one standard deviation from the mean (*N* = 3). CVs used to generate these plots were collected at a scan rate of 10 mV/s. Dotted lines at the bottom of each plot represent a current ratio corresponding to no effective catalysis.

Visualizing the substrate titration curves across the entire scope allows us to quickly assess trends and spot nonidealities in the data, as illustrated by the unexpected inhibitory behavior of GOx at increasing concentrations of d-glucose and 2-deoxy-d-glucose. Given that aerobic GOx turnover produces H_2_O_2_, which is known to inhibit GOx activity toward glucose, we hypothesized that H_2_O_2_ accumulation over the course of the multihour automated experiment was responsible for the observed deactivation. Conversely, bGOx was apparently insensitive to H_2_O_2_ or underwent a negligible competitive aerobic turnover. Guided by insight from the automated experiments, we set out to further elucidate the difference in stability between GOx and bGOx through chronoamperometry, H_2_O_2_ production studies, and H_2_O_2_ deactivation studies.

The glucose titration experiments in Figure 4a showed a decrease in electrochemical activity with an increased concentration of glucose, while bGOx displayed typical substrate-response curves. To the best of our knowledge, substrate inhibition of glucose oxidase has no precedent, implying that deactivation of the enzyme was occurring over the course of the multihour automated experiment, where each concentration point in the titration curve took roughly 10 min to obtain. As a control, we tested the response of a glucose oxidase from an alternative supplier and saw a nearly identical trend, suggesting that the inhibitory behavior was not dependent on the purity of the glucose oxidase (Figure S11).

**Figure 4:**
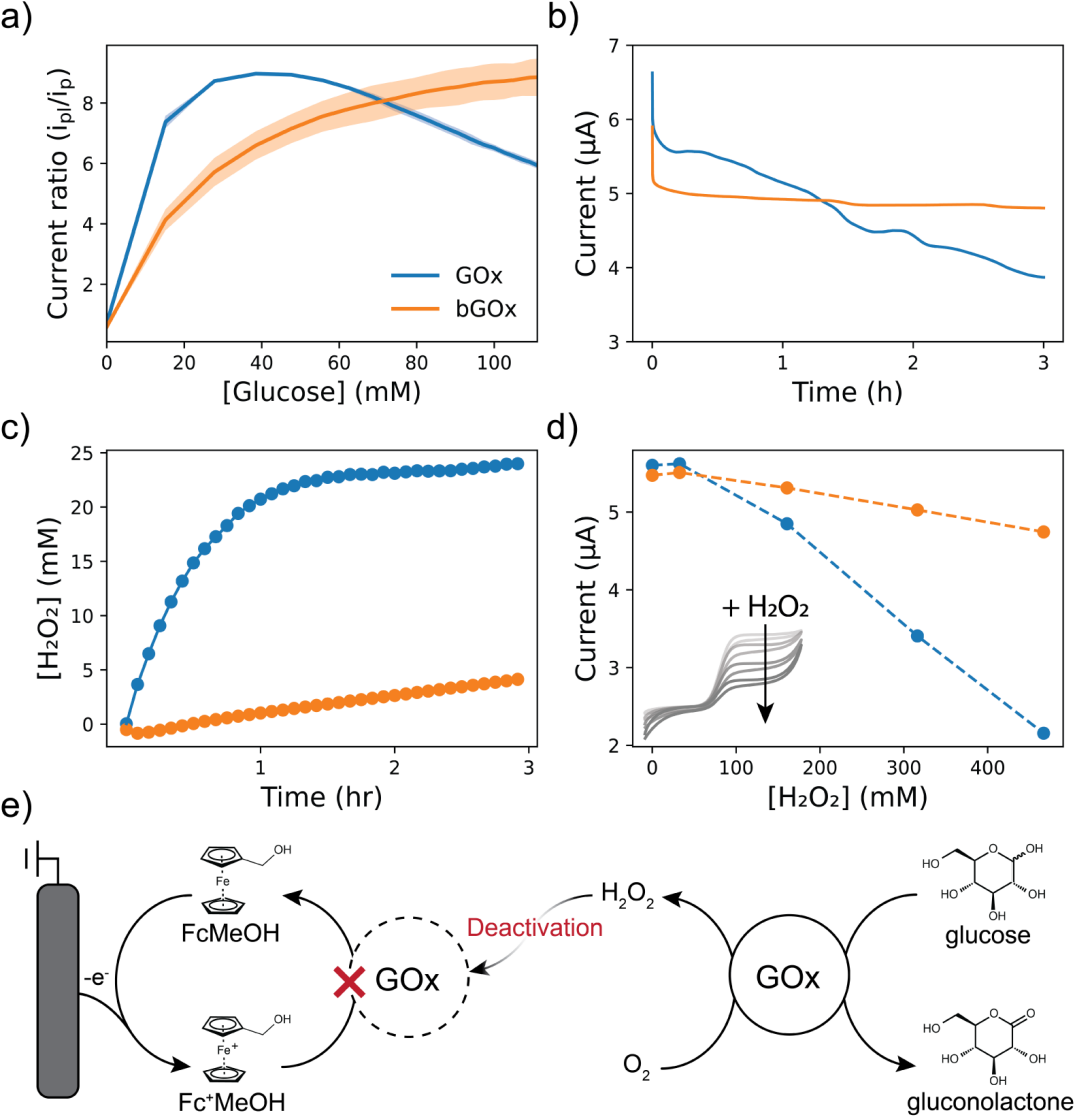
Mechanistic investigation of the role of H_2_O_2_ on enzymatic glucose oxidation. (a) Automated screening results of GOx and bGOx across glucose. (b) Chronoamperometry of 100 μM FcMeOH with 2.7 μM GOx and bGOx in the presence of 100 mM glucose. (c) H_2_O_2_ production of solely 2.7 μM GOx/bGOx in the presence of 100 mM glucose and ambient O_2_, with stirring in between CV measurements. (d) Steady-state current of 100 μM FcMeOH with 2.7 μM GOx and bGOx in the presence of 100 mM glucose, as a function of added H_2_O_2_. Inset plots show the change in GOx CVs upon the addition of H_2_O_2_. (e) Schematic depicting GOx deactivation by H_2_O_2_ produced during competitive aerobic catalysis.

We hypothesized that aerobic oxidation of GOx was competing with electrochemical turnover, leading to eventual enzyme deactivation under ambient conditions. Indeed, aerobic oxidation of GOx produces H_2_O_2_, which has been shown to inhibit GOx.^54–56^ To distinguish whether this effect was time-dependent or concentration-dependent, we ran chronoamperometry for three h at a fixed glucose concentration. Figure 4b shows the results of the chronoamperometry experiment, with GOx activity decreasing notably over the 3 h period and bGOx remaining relatively stable. The decrease in GOx activity was minimized in an oxygen-free environment (Figure S12), further verifying that the aerobic production of H_2_O_2_ was causing deactivation of GOx. From Figure 4b, we concluded that the inhibitory effects observed in the GOx titration curves were attributable to the duration of the experiment.

We proceeded to test hydrogen peroxide production over the course of three h for GOx and bGOx (Figure 4c) in the presence of 100 mM glucose. Peroxide production was measured under purely aerobic conditions, with no redox mediator and no applied potential. A platinum electrode was used to perform CV of hydrogen peroxide, and the peak current was used to calculate the concentration of peroxide in solution according to the calibration curve in Figure S13. The solution was continuously stirred between CV measurements to prevent the depletion of O_2_ over the course of the experiment, allowing for the accumulation of over 20 mM H_2_O_2_ through purely aerobic turnover (Figure S14). Wild-type GOx produces notably more peroxide than bGOx, indicating that GOx is much more reactive toward oxygen. Stabilization of O_2_^–^ by a protonated histidine residue in the GOx active site (His-516) has been shown to drive GOx reaction with O_2_ through site-directed mutagenesis and extensive kinetic studies.^57^ Although the exact structure is unknown to us, we posit that such residues are not conserved in the bGOx variant, dramatically impacting aerobic production of H_2_O_2_.

Finally, we performed CVs with intentionally added H_2_O_2_ to further confirm that it was the aerobically formed H_2_O_2_ that was responsible for GOx deactivation, which is well supported by previous literature.^54–56^ Figure 4d shows how the steady-state plateau current (10 mV/s scan rate, 2.7 μM enzyme, 100 μM FcMeOH, and 100 mM glucose in 100 mM KPi) decreased upon the addition of increasing concentrations of H_2_O_2_. The GOx steady-state current was 39% of its initial value after reaching concentrations of ∼466 mM H_2_O_2_, while the bGOx steady-state current was still 87% of its initial value. Such a marked decrease in the activity of GOx is consistent with reports in the literature, which implicate the oxidation of methionine residues to methionine sulfoxide by H_2_O_2_ as a possible pathway of GOx deactivation.^55^ An overall schematic depicting H_2_O_2_ induced deactivation and its impact on the electrochemical turnover of GOx is shown in Figure 4e. We note that the stability of bGOx is in line with other glucose oxidase variants that can tolerate oligosaccharide substrates^56^ and is highly beneficial for developing enzymatic electrochemical devices that can tolerate practical conditions.^58^

## Conclusions

In this work, automated electrochemistry was used to screen the substrate-dependent activity of GOx and bGOx-mediated electrocatalytic systems. We performed over 10,000 CV measurements across a combination of substrates, enzymes, concentrations, and scan rates. The insight from our automated campaign led to our hypothesis that aerobic production of H_2_O_2_ was a key contributor to GOx deactivation, and the promiscuous variant bGOx was relatively insensitive to H_2_O_2_. We further confirmed this through manual experimentation, confirming our hypothesis. We highlight our use of automated experiments to efficiently generate chemical insight as a key takeaway from this work, a paradigm we have employed in our studies of other bioelectrochemical systems.^26^

We envision the eLab platform, which was replicated in under a day of effort, will enable further high-throughput experimental screening campaigns to identify chemical insight across enzyme variant, enzyme-substrate, and enzyme-mediator libraries.^10,59,60^ The enhanced throughput of our automated electrochemistry platform allows for the screening of engineered redox-active enzymes^19,61–63^ and will assist in the genetic engineering of novel enzyme variants with tailored selectivity and practical operating conditions.^64,65^ Incorporating parallel multipotentiostat platforms such as LEGION^66,67^ and machine-learning-accelerated CV investigations^46,68^ will further expand the throughput of automated electrochemical analysis, enabling directed evolution and de novo design campaigns targeting non-native enzyme functionality.^23,69,70^ This work represents an important interdisciplinary effort blending electrochemistry, biology, and automation, and we hope that the demonstrated, accessible workflow will help lower the barrier to high-throughput enzymatic electrochemistry.

## Supplementary Material



## Data Availability

All data and code used in this work are made publicly available in the following Zenodo repository: https://doi.org/10.5281/zenodo.18897022

## Notes

Schematics of the robotic device and additional supporting cyclic voltammograms (PDF)

## ∥

Baylor University (Department of Chemistry)
