# Supplementary material for: An Automated Electrochemistry Platform for Accelerating the Characterization of Enzymatic Electrochemistry
Source: ACS Electrochem. 2026 Apr 29;2(7):1519–26. doi: 10.1021/acselectrochem.6c00095 (PMC13339139; doi:10.1021/acselectrochem.6c00095)
Supplement: Supplementary file 1 [file ec6c00095_si_001.pdf]

Supporting Information

An Automated Electrochemistry Platform for Accelerating the Characterization of Enzymatic Electrochemistry

Michael A. Pence[1], Zachary A. Nguyen[2], Luke G. Kays[2], Dylan G. Boucher*[,2,‡], Joaquín Rodríguez-López*[,3], Shelley D. Minteer*[,1]

1. *Missouri University of Science and Technology (Department of Chemistry and Kummer Institute Center for Resource Sustainability)*
2. *University of Utah (Department of Chemistry)*
3. *University of Illinois Urbana-Champaign (Department of Chemistry and Beckman Institute)*

‡ Now at Baylor University (Department of Chemistry)
** Corresponding author*

D.G.B. email: Dylan_Boucher@baylor.edu
J.R.L. email: joaquinr@illinois.edu
S.D.M. email: shelley.minteer@mst.edu

Table of Contents

Figure S1. Schematic and picture of the automated electrochemistry platform. S2

Figure S2. Schematic for the automated stir plate. S2

Figure S3. Effect of scan rate on CVs. S3

Note S1. Calculating k_{obs} from peak ratios. S3

Figure S4. Example of fitting data to the Michaelis-Menten equation. S3

Figure S5. Selected triplicate CV data for bGOx. S4

Figure S6. Selected triplicate CV data for GOx. S5

Figure S7. Comparison of FcMeOH peak currents across replicates. S6

Figure S8. CVs of representative sugars S6

Figure S9. CV behavior of FcMeOH. S6

Figure S10. Effect of scan rate on current ratio vs. concentration curves. S7

Figure S11. Comparison of bGOx and GOx from two different suppliers. S7

Figure S12. Comparison of GOx instability in ambient and oxygen-free conditions. S7

Figure S13. Construction of an H_2O_2 calibration curve. S8

Figure S14. Qualitative assessment of generated H_2O_2. S8

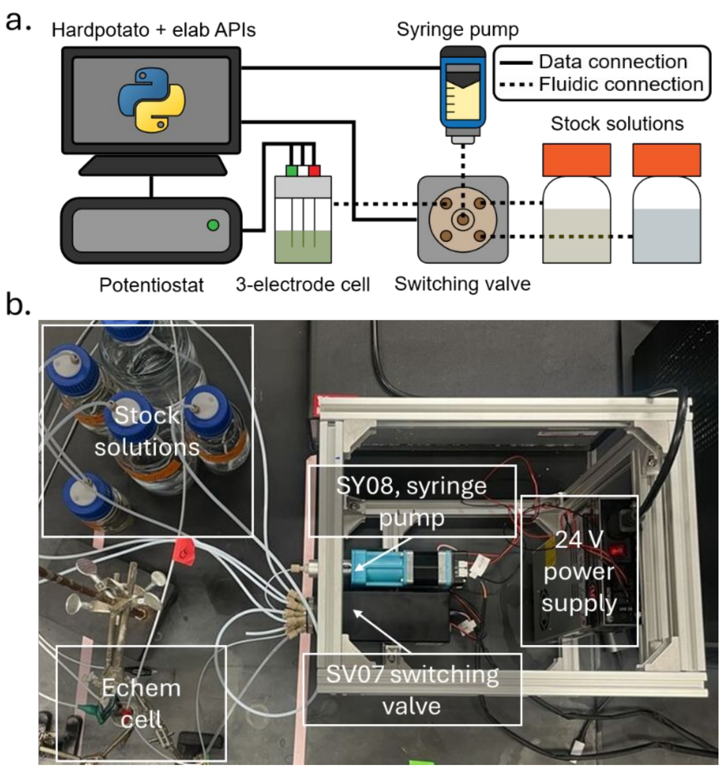

Figure S1. *(a) Schematic and (b) picture of the automated electrochemistry platform with major components labeled.*

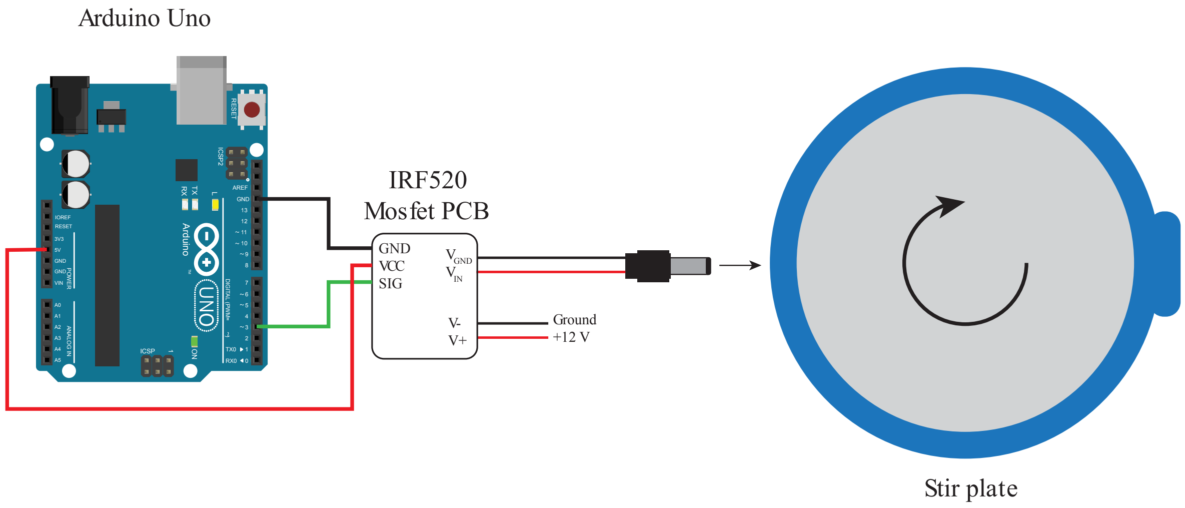

Figure S2. Schematic for assembling the automated stir plate circuit. Components needed include a 12 V power supply, and Arduino, an IRF520 MOSFET breakout board PCB, and a barrel jack for connecting to the stir plate (here an IKA Big Squid).

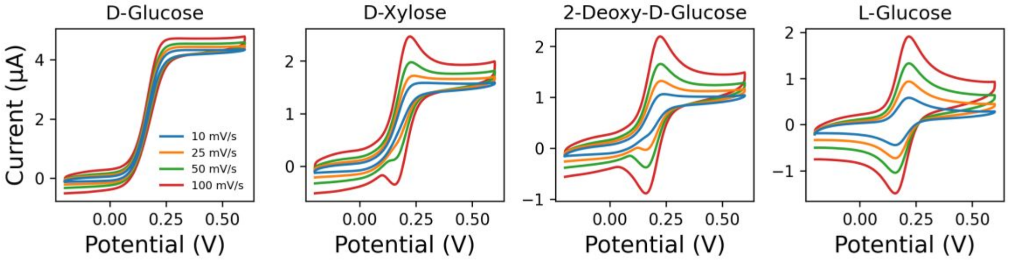

***Figure S3.** Effect of scan rate on CVs. CVs of 0.1 mM FcMeOH with 2.7 μM bGOx with 100 mM of four different substrates (D-glucose, D-xylose, 2-deoxy-D-glucose, L-glucose) at all measured scan rates. True steady state measurements, i.e. those of D-glucose, are unaffected by changing scan rate.*

Note S1.

The ratio between the CV plateau current in the presence of substrate, i_{pl}, and the peak current in the absence of substrate, i_p, can be converted to kinetic parameters through the following equation

$$\frac{i_{pl}}{i_p} = \frac{n}{0.4463n'}\sqrt{\frac{RTk_{obs}}{n'Fv}}$$

where F is Faraday's constant, R is the ideal gas constant, T is temperature, n is the number of electrons transferred in the catalytic cycle, n' is the number of electrons transferred to the catalyst, and k_{obs} is the observed rate constant.

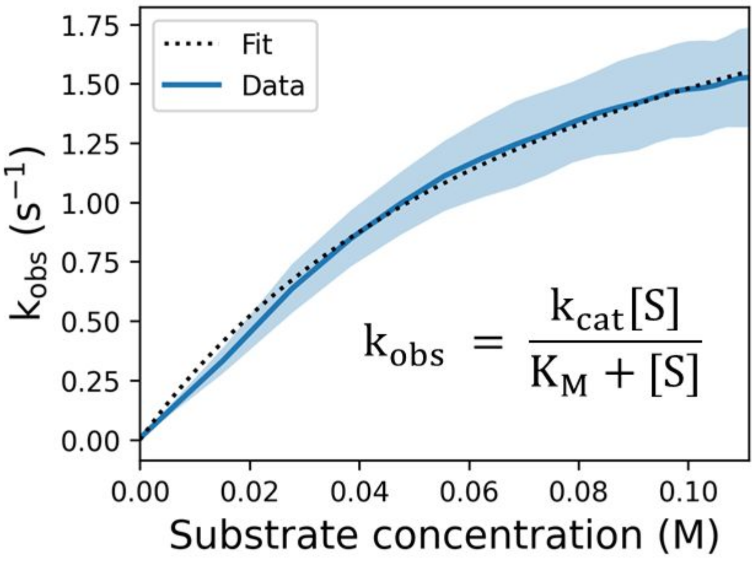

Figure S4. *Example of the Michaelis-Menten equation fit to the observed rate constant as a function of glucose concentration in 2.7 μM bGOx and 100 μM FcMeOH in KPi buffer. The calculated K_M is 85 mM and the calculated k_{cat} is 2.7 s^{-1}.*

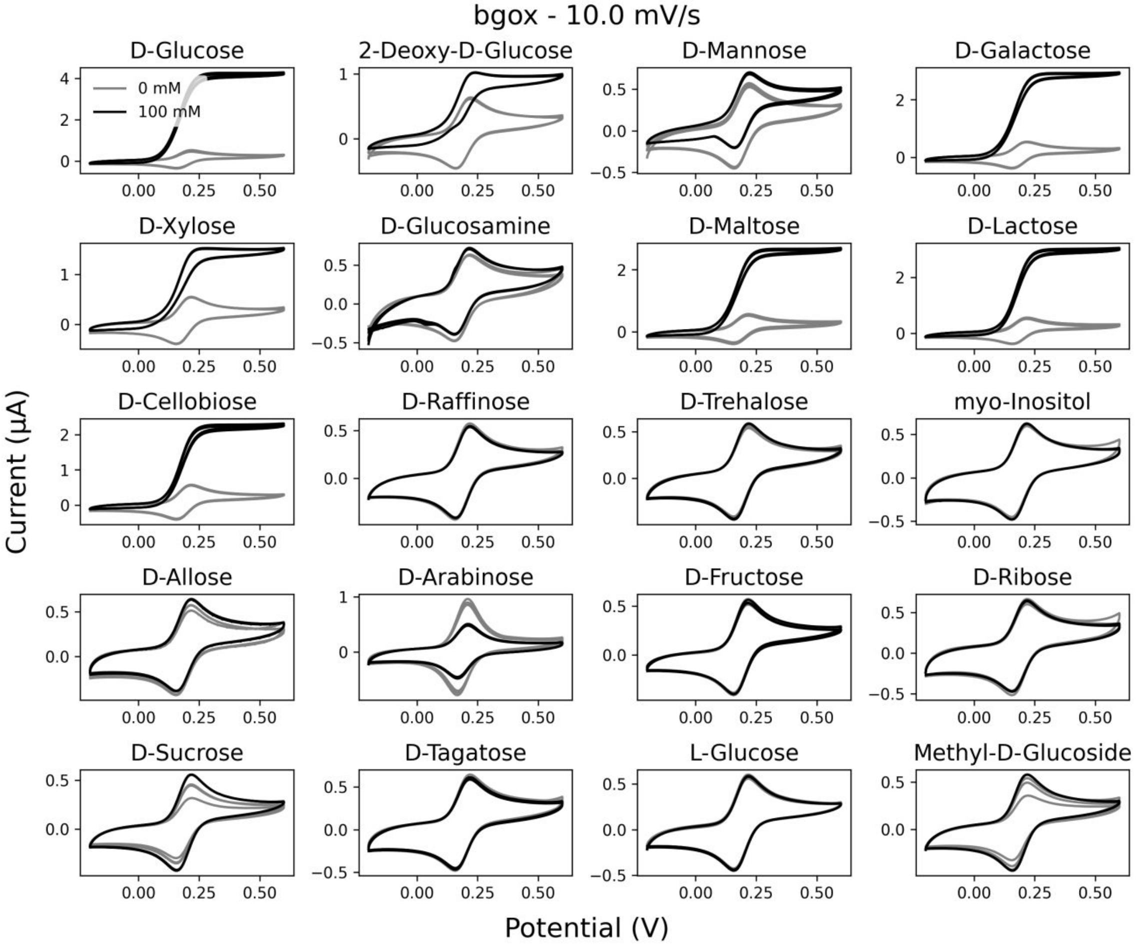

Figure S5. *Triplicate data for CVs of 2.7 μM bGOx, 100 μM FcMeOH, and either 0 mM (grey) or 100 mM (black) of the labeled substrate. All CVs were collected at 10 mV/s.*

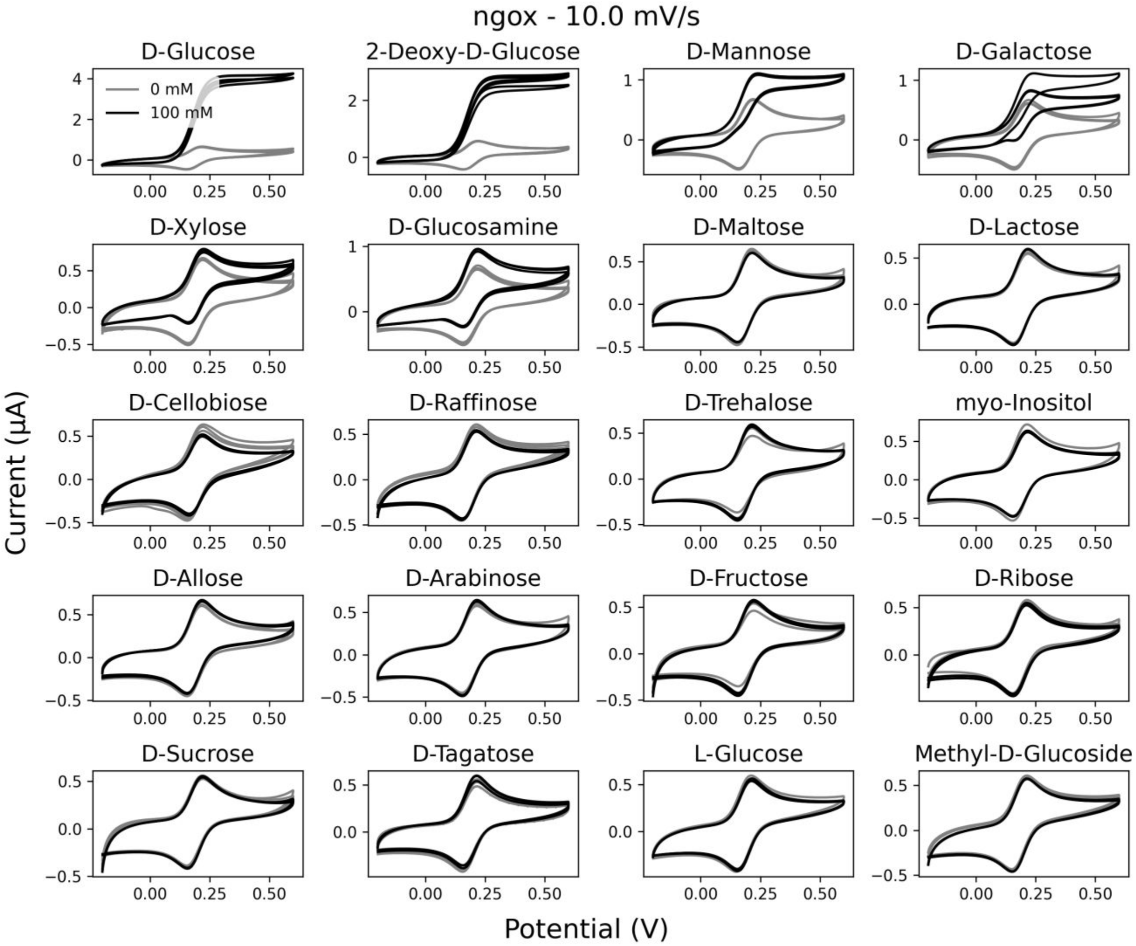

Figure S6. *Triplicate data for CVs of 2.7 μM GOx, 100 μM FcMeOH, and either 0 mM (grey) or 100 mM (black) of the labeled substrate. All CVs were collected at 10 mV/s.*

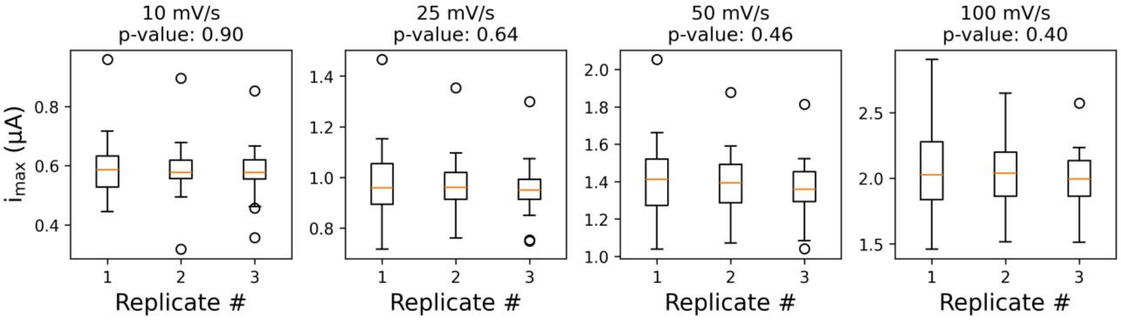

Figure S7. *Comparison of peak currents obtained from CVs of 2.7 µM enzyme and 100 µM FcMeOH in 100 mM KPi buffer across the differedifferent, at all scan rates. One-way ANOVA shows no significant difference across the replicates.*

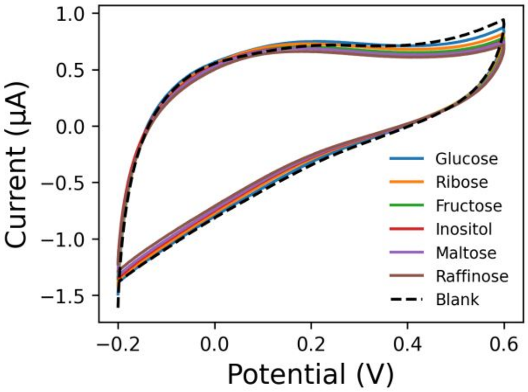

Figure S8. *Cyclic voltammograms of 100 mM of representative sugars in 100 mM KPi buffer, demonstrating no distinct redox activity in the potential window used in this work.*

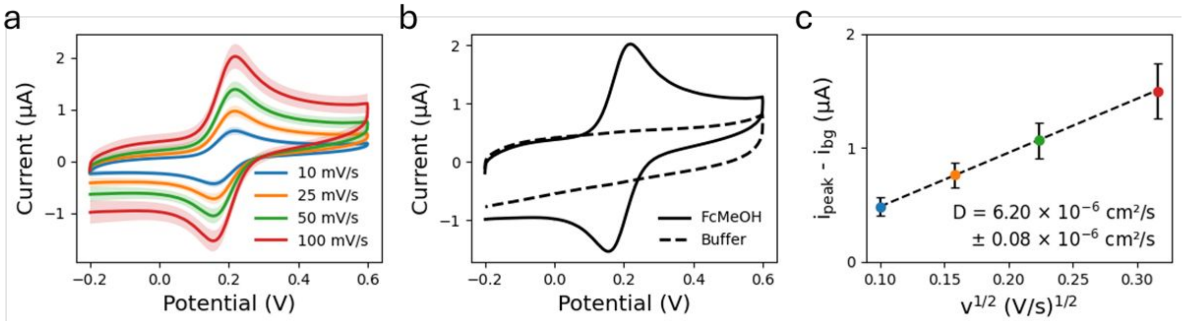

Figure S9. *CV behavior of FcMeOH in the absence of substrate. (a) Average CVs of 2.7 µM enzyme and 100 µM FcMeOH in KPi buffer collected at scan rates of 10, 25, 50, and 100 mV/s. Error bands represent 1 standard deviation from the mean. (b) Comparison of CVs of 2.7 µM enzyme in KPi buffer with and without 100 µM FcMeOH, showing the notable non-faradaic current response. Due to the significant non faradaic current, background subtracted peak currents were used to calculate diffusion coefficient. (c) Average anodic peak current as a function of the square root of scan rate. Feeding the fitted slope into the Randles–Ševčík equation yields a diffusion coefficient of 6.20 × 10^{-6} cm^2/s with a standard deviation of 0.08 × 10^{-6} cm^2/s.*

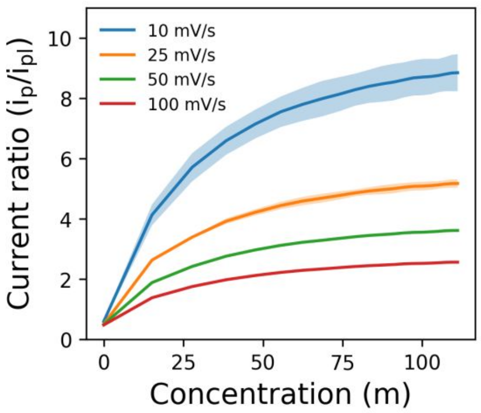

Figure S10. *Plot of the peak/plateau current ratio at different scan rates as a function of glucose concentration in 2.7 µM bGOx and 100 µM FcMeOH in KPi buffer.*

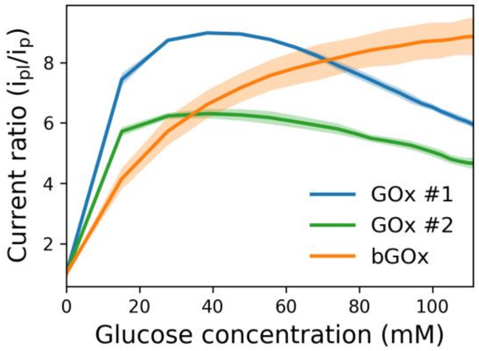

Figure S11. *Plot of the peak/plateau current ratio as a function of glucose concentration in 2.7 µM enzyme and 100 µM FcMeOH in KPi buffer. Data shown is extracted from CVs taken at a scan rate of 10 mV/s. GOx #1 was obtained from Sigma Aldrich and GOx #2 was obtained from Amano. Both show a diminished effect at higher glucose concentrations.*

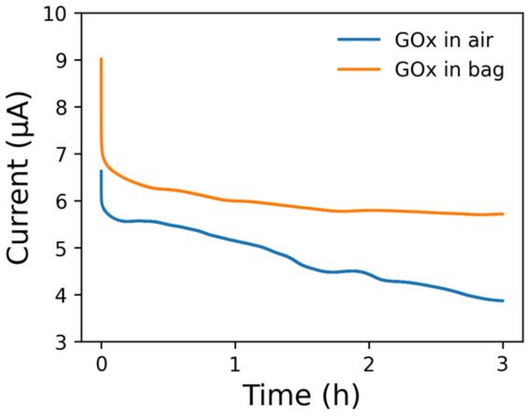

Figure S12. *Comparison of amperometry of 2.7 µM GOx, 100 µM FcMeOH, and 100 mM of glucose in ambient air (blue) and in an oxygen free glove-bag (orange). Chronoamperometry was performed at a diffusion-limited potential of 0.6 V vs. SCE for a total of 3 hr.*

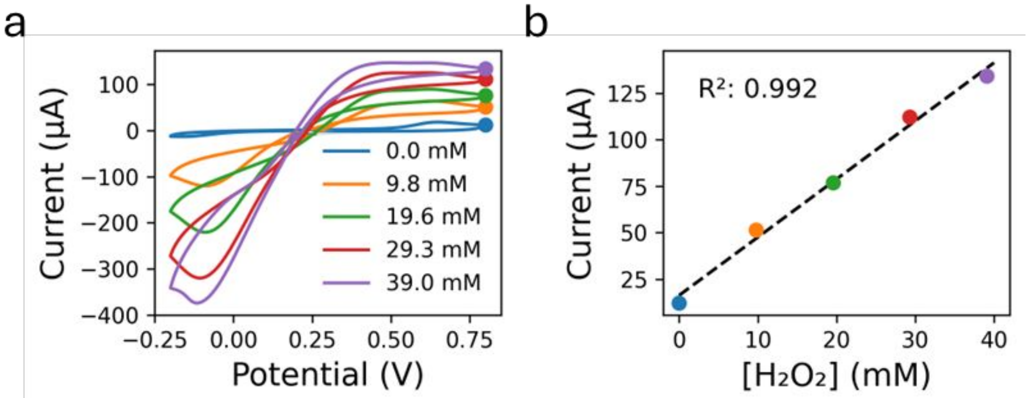

Figure S13. *Developing a H_2O_2 calibration curve.* ***(a)*** *CVs of different concentrations of H_2O_2 in KPi buffer. Dots on the CV at 0.8 V vs. SCE represent where the current was sampled for construction of the calibration curve. CVs were taken at a 2 mm Pt working electrode.* ***(b)*** *Calibration curve constructed from the data shown in* ***(a).***

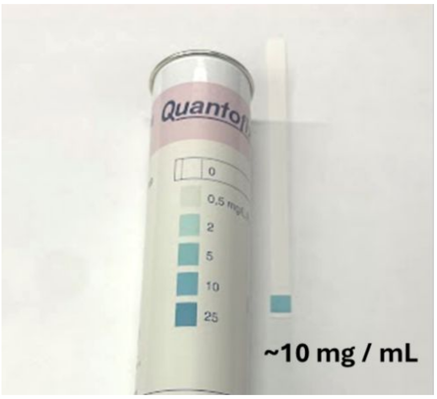

Figure S14. *Qualitative measurement with peroxide test strips of the 100-fold diluted product resulting from mixing 2.7 μM GOx and 100 mM glucose while stirring for three hours, with the estimated concentration of peroxide produced being ~29 mM.*
